# *catena*-Poly[[aqua­(imidazole)­cadmium(II)]-μ_3_-benzene-1,3-dicarboxyl­ato]

**DOI:** 10.1107/S1600536810022117

**Published:** 2010-06-16

**Authors:** Zhengfang Zeng, Hongyan Xu

**Affiliations:** a School of Sciences, Nanchang University, Nanchang, Jiangxi 330031, People’s Republic of China; b Fujian Institute of Research on the Structure of Matter, Chinese Academy of Sciences, Fuzhou, Fujian 350002, People’s Republic of China

## Abstract

In the title compound, [Cd(C_8_H_4_O_4_)(C_3_H_4_N_2_)(H_2_O)]_*n*_, the Cd^II^ ion is seven-coordinated by five O atoms from three crystallographically independent benzene-1,3-carboxylate ligands, one N  atom from the imidazole ligand and one coordinated water mol­ecule. Neighboring Cd^II^ ions are bridged by the benzene-1,3-dicarboxyl­ate ligands, forming a zigzag polymeric chain structure. These chains are further extended into a three-dimensional supra­molecular structure through O—H⋯O and N—H⋯O hydrogen bonds.

## Related literature

For the synthesis, see: Yaghi *et al.* (1998 ▶). For related structures, see: Ma *et al.* (2008 ▶); Wang *et al.* (2008 ▶).
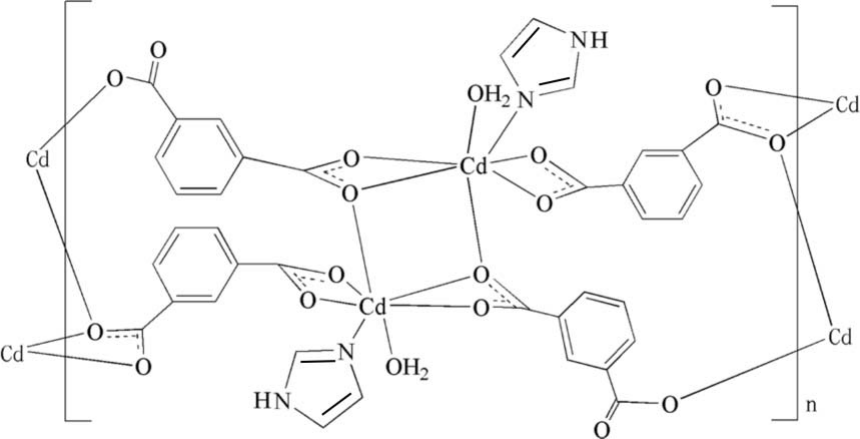


## Experimental

### 

#### Crystal data

[Cd(C_8_H_4_O_4_)(C_3_H_4_N_2_)(H_2_O)]
*M* *_r_* = 362.61
Triclinic, 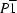

*a* = 8.2616 (8) Å
*b* = 8.3138 (8) Å
*c* = 10.235 (1) Å
α = 67.017 (2)°
β = 68.176 (2)°
γ = 81.054 (2)°
*V* = 600.76 (10) Å^3^
*Z* = 2
Mo *K*α radiation
μ = 1.84 mm^−1^
*T* = 293 K
0.27 × 0.18 × 0.06 mm

#### Data collection

Bruker APEX CCD diffractometer
Absorption correction: multi-scan (*SADABS*; Sheldrick, 1996 ▶) *T*_min_ = 0.536, *T*_max_ = 1.000
3166 measured reflections
2086 independent reflections
2049 reflections with *I* > 2σ(*I*)
*R*_int_ = 0.019

#### Refinement

*R*[*F* ^2^ > 2σ(*F* ^2^)] = 0.028
*wR*(*F* ^2^) = 0.072
*S* = 1.15
2086 reflections
184 parameters
H atoms treated by a mixture of independent and constrained refinement
Δρ_max_ = 0.41 e Å^−3^
Δρ_min_ = −0.79 e Å^−3^

### 

Data collection: *SMART* (Siemens, 1996 ▶); cell refinement: *SAINT* (Siemens, 1996 ▶); data reduction: *SAINT*; program(s) used to solve structure: *SHELXS97* (Sheldrick, 2008 ▶); program(s) used to refine structure: *SHELXL97* (Sheldrick, 2008 ▶); molecular graphics: *SHELXTL* (Sheldrick, 2008 ▶); software used to prepare material for publication: *SHELXTL*.

## Supplementary Material

Crystal structure: contains datablocks I, global. DOI: 10.1107/S1600536810022117/lx2148sup1.cif

Structure factors: contains datablocks I. DOI: 10.1107/S1600536810022117/lx2148Isup2.hkl

Additional supplementary materials:  crystallographic information; 3D view; checkCIF report

## Figures and Tables

**Table 1:** Selected bond lengths (Å)

Cd1—N1	2.216 (3)
Cd1—O3^i^	2.251 (3)
Cd1—O2^ii^	2.311 (3)
Cd1—O5	2.394 (3)
Cd1—O1	2.413 (3)
Cd1—O1^ii^	2.626 (3)
Cd1—O4^i^	2.663 (3)

Symmetry codes: (i) 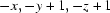; (ii) 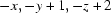.

**Table 2:** Hydrogen-bond geometry (Å, °)

*D*—H⋯*A*	*D*—H	H⋯*A*	*D*⋯*A*	*D*—H⋯*A*
O5—H5*A*⋯O2^iii^	0.84 (8)	2.07 (8)	2.809 (4)	146 (7)
O5—H5*B*⋯O3^iv^	0.87 (8)	1.96 (8)	2.786 (4)	158 (7)
N2—H2⋯O4^v^	0.85 (7)	2.00 (7)	2.854 (5)	175 (6)

Symmetry codes: (iii) 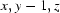; (iv) 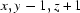; (v) 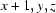.

**Fig. 1.**
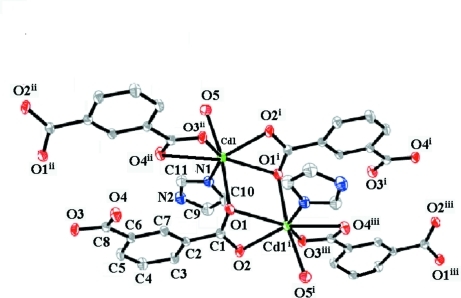
The molecular structure of (I), with atom labels and 50% probability displacement ellipsoids for non-H atoms. [Symmetry codes: (i) -x, -y+1, -z+2; (ii) -x, -y+1, -z+1; (iii) x, y, z+1.]

**Fig. 2.**
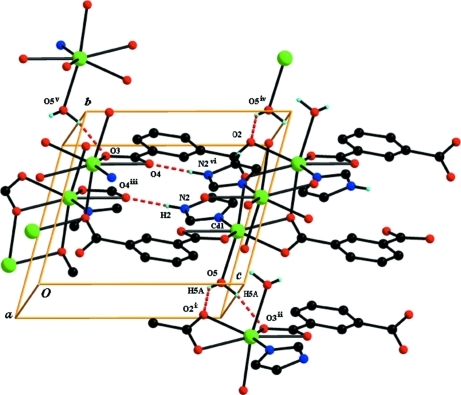
The crystal packing of (I), showing one layer of molecules connected by O–H···O hydrogen bonds (dashed lines). H atoms not involved in hydrogen bonding have been omitted. [Symmetry codes: (i) x, y-1 , z; (ii) x, y-1, z+1; (iii) x+1, y, z; (iv) -x, -y+1, -z+1; (v) -x, -y+1, -z+2.]

## supplementary crystallographic information

### Comment

In recent years, considerable effort was paied in the study of metal-organic hybrid materials with the extended supermolecular framework structures, owing to their intriguing network topologies and potential application in adsorption, molecular recognition, catalysis and maganetism (Yaghi *et al.*, 1998 ). Currently, carboxylic acids have been widely used as polydentate and bridging ligands since they can bind metal ions in diverse modes and play an important role in adjusting verious topologies structures. A number of promising supramolecular complexes have been designed and constructed by using carboxylic acids as favorable conditions. (Ma *et al.*, 2008; Wang *et al.*, 2008). In this paper, we report the synthesis and structural characterzation of the title compound in which the isophthalic acid ligand displayed its good coordination ability and diverse coordination modes.

The Cd^II^ ion is six-coordinated by four O atoms from three crystallographically independent benzene-1,3- dicarboxylate ligands and one N atom from the chelating imidazole ligand and one coordinated water molecule (Fig. 1 & Table 1). The neighboring Cd^i^ ion is bridged by the benzene-1, 3-dicarboxylate ligands to form a one-dimensional chain structure. In the crystal structure, the adjacent chains are linked *via* O–H···O and N–H···O hydrogen bonds (Fig. 2 & Table 2) resulting in the formation of a three dimensional supramolecular structure.

### Experimental

A 10 ml aqueous solution of imidazole (0.014 g, 0.20 mmol) and isophthalic acid (0.034 g,0.020 *m*mol) was slowly added into cadmium nitrate (0.062 g, 0.20 *m*mol) solution in methanol (10 ml), and the mixed solution was stirred for 20 min and then was heated in a 30 ml Teflon-line autoclave under autogeneous pressure at 423 K for 3 d. After cooling to room temperature, colorless block crystals were obtained. (yield 38%).

### Refinement

H atoms bound to H_2_O and NH atoms were located in a difference map and refined freely. Other H atoms were positioned geometrically and refined using a riding model, with C–H = 0.93 Å and *U*_iso_(H) = 1.2*U*_eq_(C).

### Crystal data

**Table:**

[Cd(C_8_H_4_O_4_)(C_3_H_4_N_2_)(H_2_O)]	*Z* = 2
*M**_r_* = 362.61	*F*(000) = 356
Triclinic, *P*1	*D*_x_ = 2.005 Mg m^−^^3^
Hall symbol: -P 1	Mo *K*α radiation, λ = 0.71073 Å
*a* = 8.2616 (8) Å	Cell parameters from 3126 reflections
*b* = 8.3138 (8) Å	θ = 3.3–27.5°
*c* = 10.235 (1) Å	µ = 1.84 mm^−^^1^
α = 67.017 (2)°	*T* = 293 K
β = 68.176 (2)°	Block, yellow
γ = 81.054 (2)°	0.27 × 0.18 × 0.06 mm
*V* = 600.76 (10) Å^3^	

### Data collection

**Table:**

Bruker APEX CCD diffractometer	2086 independent reflections
Radiation source: fine-focus sealed tube	2049 reflections with *I* > 2σ(*I*)
graphite	*R*_int_ = 0.019
Detector resolution: 10 pixels mm^-1^	θ_max_ = 25.0°, θ_min_ = 2.3°
ω scans	*h* = −9→9
Absorption correction: multi-scan (*SADABS*; Sheldrick, 1996)	*k* = −9→4
*T*_min_ = 0.536, *T*_max_ = 1.000	*l* = −12→11
3166 measured reflections	

### Refinement

**Table:**

Refinement on *F*^2^	Primary atom site location: structure-invariant direct methods
Least-squares matrix: full	Secondary atom site location: difference Fourier map
*R*[*F*^2^ > 2σ(*F*^2^)] = 0.028	Hydrogen site location: difference Fourier map
*wR*(*F*^2^) = 0.072	H atoms treated by a mixture of independent and constrained refinement
*S* = 1.15	*w* = 1/[σ^2^(*F*_o_^2^) + (0.0315*P*)^2^ + 1.1125*P*] where *P* = (*F*_o_^2^ + 2*F*_c_^2^)/3
2086 reflections	(Δ/σ)_max_ < 0.001
184 parameters	Δρ_max_ = 0.41 e Å^−^^3^
0 restraints	Δρ_min_ = −0.79 e Å^−^^3^

### Special details

**Table:**

Geometry. All esds (except the esd in the dihedral angle between two l.s. planes) are estimated using the full covariance matrix. The cell esds are taken into account individually in the estimation of esds in distances, angles and torsion angles; correlations between esds in cell parameters are only used when they are defined by crystal symmetry. An approximate (isotropic) treatment of cell esds is used for estimating esds involving l.s. planes.
Refinement. Refinement of F^2^ against ALL reflections. The weighted R-factor wR and goodness of fit S are based on F^2^, conventional R-factors R are based on F, with F set to zero for negative F^2^. The threshold expression of F^2^ > 2sigma(F^2^) is used only for calculating R-factors(gt) etc. and is not relevant to the choice of reflections for refinement. R-factors based on F^2^ are statistically about twice as large as those based on F, and R- factors based on ALL data will be even larger.

### Fractional atomic coordinates and isotropic or equivalent isotropic displacement parameters (Å^2^)

**Table:**

	*x*	*y*	*z*	*U*_iso_*/*U*_eq_	
Cd1	0.08672 (3)	0.32297 (3)	0.90289 (3)	0.02898 (11)	
O1	−0.0532 (4)	0.6070 (4)	0.8593 (3)	0.0337 (6)	
O2	0.0672 (4)	0.8334 (4)	0.8475 (3)	0.0382 (6)	
O3	0.1082 (4)	0.7617 (4)	0.1628 (3)	0.0347 (6)	
O4	−0.1232 (4)	0.6718 (4)	0.3685 (3)	0.0405 (7)	
O5	0.2389 (4)	0.0533 (4)	0.9029 (4)	0.0414 (7)	
H5A	0.209 (10)	0.017 (10)	0.849 (9)	0.10 (2)*	
H5B	0.211 (10)	−0.024 (10)	0.994 (9)	0.10 (3)*	
N1	0.3422 (4)	0.4507 (4)	0.8114 (4)	0.0340 (7)	
N2	0.5999 (5)	0.5524 (5)	0.6516 (4)	0.0453 (9)	
H2	0.687 (9)	0.587 (9)	0.570 (8)	0.08 (2)*	
C1	0.0393 (5)	0.7429 (5)	0.7840 (4)	0.0272 (7)	
C2	0.1174 (5)	0.7962 (5)	0.6165 (4)	0.0255 (7)	
C3	0.2660 (5)	0.8979 (5)	0.5366 (4)	0.0325 (8)	
H3	0.3153	0.9372	0.5868	0.039*	
C4	0.3406 (5)	0.9406 (6)	0.3814 (5)	0.0398 (9)	
H4	0.4428	1.0048	0.3284	0.048*	
C5	0.2642 (5)	0.8885 (5)	0.3047 (4)	0.0333 (8)	
H5	0.3145	0.9184	0.2006	0.040*	
C8	0.0251 (5)	0.7384 (5)	0.3016 (4)	0.0293 (8)	
C7	0.0420 (4)	0.7439 (4)	0.5394 (4)	0.0248 (7)	
H7	−0.0575	0.6757	0.5930	0.030*	
C11	0.4652 (6)	0.4570 (6)	0.6844 (5)	0.0425 (10)	
H11	0.4592	0.4010	0.6235	0.051*	
C10	0.4032 (6)	0.5481 (6)	0.8641 (5)	0.0436 (10)	
H10	0.3441	0.5688	0.9532	0.052*	
C9	0.5632 (6)	0.6097 (7)	0.7663 (6)	0.0488 (11)	
H9	0.6338	0.6779	0.7762	0.059*	
C6	0.1121 (5)	0.7914 (5)	0.3837 (4)	0.0261 (7)	

### Atomic displacement parameters (Å^2^)

**Table:**

	*U*^11^	*U*^22^	*U*^33^	*U*^12^	*U*^13^	*U*^23^
Cd1	0.03179 (17)	0.03293 (17)	0.02429 (16)	−0.01047 (11)	−0.00754 (11)	−0.01069 (12)
O1	0.0381 (15)	0.0359 (15)	0.0243 (13)	−0.0070 (12)	−0.0089 (11)	−0.0074 (11)
O2	0.0528 (17)	0.0447 (16)	0.0260 (13)	−0.0095 (13)	−0.0144 (12)	−0.0175 (12)
O3	0.0442 (15)	0.0420 (15)	0.0236 (13)	−0.0069 (12)	−0.0114 (11)	−0.0154 (11)
O4	0.0422 (16)	0.0530 (18)	0.0302 (14)	−0.0195 (13)	−0.0118 (12)	−0.0125 (13)
O5	0.0440 (17)	0.0385 (16)	0.0405 (17)	−0.0083 (13)	−0.0105 (14)	−0.0137 (15)
N1	0.0310 (16)	0.0374 (18)	0.0328 (17)	−0.0080 (13)	−0.0085 (13)	−0.0114 (14)
N2	0.0336 (19)	0.055 (2)	0.039 (2)	−0.0110 (17)	−0.0046 (16)	−0.0120 (18)
C1	0.0274 (18)	0.0310 (19)	0.0236 (17)	−0.0004 (14)	−0.0103 (14)	−0.0091 (15)
C2	0.0289 (18)	0.0254 (17)	0.0223 (17)	−0.0011 (14)	−0.0100 (14)	−0.0073 (14)
C3	0.036 (2)	0.042 (2)	0.0276 (19)	−0.0122 (16)	−0.0133 (16)	−0.0137 (16)
C4	0.033 (2)	0.053 (3)	0.032 (2)	−0.0214 (18)	−0.0048 (16)	−0.0118 (18)
C5	0.035 (2)	0.042 (2)	0.0202 (17)	−0.0076 (16)	−0.0060 (15)	−0.0092 (16)
C8	0.038 (2)	0.0273 (18)	0.0267 (18)	−0.0028 (15)	−0.0152 (16)	−0.0089 (15)
C7	0.0250 (17)	0.0278 (17)	0.0220 (17)	−0.0045 (13)	−0.0084 (13)	−0.0075 (14)
C11	0.040 (2)	0.050 (3)	0.040 (2)	−0.0124 (19)	−0.0100 (18)	−0.017 (2)
C10	0.036 (2)	0.057 (3)	0.042 (2)	−0.0128 (19)	−0.0100 (18)	−0.021 (2)
C9	0.040 (2)	0.057 (3)	0.054 (3)	−0.017 (2)	−0.015 (2)	−0.020 (2)
C6	0.0285 (18)	0.0281 (18)	0.0248 (17)	−0.0013 (14)	−0.0113 (14)	−0.0105 (14)

### Geometric parameters (Å, °)

**Table:**

Cd1—N1	2.216 (3)	N2—H2	0.85 (7)
Cd1—O3^i^	2.251 (3)	C1—C2	1.492 (5)
Cd1—O2^ii^	2.311 (3)	C2—C7	1.384 (5)
Cd1—O5	2.394 (3)	C2—C3	1.388 (5)
Cd1—O1	2.413 (3)	C3—C4	1.389 (5)
Cd1—O1^ii^	2.626 (3)	C3—H3	0.9300
Cd1—O4^i^	2.663 (3)	C4—C5	1.387 (6)
O1—C1	1.266 (5)	C4—H4	0.9300
O2—C1	1.260 (4)	C5—C6	1.391 (5)
O3—C8	1.276 (4)	C5—H5	0.9300
O4—C8	1.252 (5)	C8—C6	1.501 (5)
O5—H5A	0.84 (8)	C7—C6	1.387 (5)
O5—H5B	0.87 (8)	C7—H7	0.9300
N1—C11	1.306 (5)	C11—H11	0.9300
N1—C10	1.368 (5)	C10—C9	1.356 (6)
N2—C11	1.330 (6)	C10—H10	0.9300
N2—C9	1.351 (6)	C9—H9	0.9300
			
N1—Cd1—O3^i^	143.34 (11)	C9—N2—H2	124 (4)
N1—Cd1—O2^ii^	127.43 (11)	O2—C1—O1	121.5 (3)
O3^i^—Cd1—O2^ii^	88.49 (10)	O2—C1—C2	119.4 (3)
N1—Cd1—O5	88.63 (11)	O1—C1—C2	119.1 (3)
O3^i^—Cd1—O5	87.33 (11)	C7—C2—C3	119.4 (3)
O2^ii^—Cd1—O5	85.20 (11)	C7—C2—C1	120.0 (3)
N1—Cd1—O1	89.20 (11)	C3—C2—C1	120.5 (3)
O3^i^—Cd1—O1	89.03 (10)	C2—C3—C4	119.7 (3)
O2^ii^—Cd1—O1	103.25 (10)	C2—C3—H3	120.2
O5—Cd1—O1	170.71 (11)	C4—C3—H3	120.2
N1—Cd1—O1^ii^	83.08 (10)	C5—C4—C3	120.6 (4)
O3^i^—Cd1—O1^ii^	131.61 (9)	C5—C4—H4	119.7
O2^ii^—Cd1—O1^ii^	52.58 (9)	C3—C4—H4	119.7
O5—Cd1—O1^ii^	112.79 (11)	C4—C5—C6	119.8 (3)
O1—Cd1—O1^ii^	75.89 (9)	C4—C5—H5	120.1
N1—Cd1—O4^i^	91.00 (10)	C6—C5—H5	120.1
O3^i^—Cd1—O4^i^	52.54 (9)	O4—C8—O3	121.5 (3)
O2^ii^—Cd1—O4^i^	137.72 (9)	O4—C8—C6	120.7 (3)
O5—Cd1—O4^i^	78.09 (11)	O3—C8—C6	117.8 (3)
O1—Cd1—O4^i^	92.92 (9)	C2—C7—C6	121.2 (3)
O1^ii^—Cd1—O4^i^	167.35 (9)	C2—C7—H7	119.4
C1—O1—Cd1	119.5 (2)	C6—C7—H7	119.4
C1—O1—Cd1^ii^	85.5 (2)	N1—C11—N2	112.7 (4)
Cd1—O1—Cd1^ii^	104.11 (9)	N1—C11—H11	123.7
C1—O2—Cd1^ii^	100.4 (2)	N2—C11—H11	123.7
C8—O3—Cd1^i^	102.2 (2)	C9—C10—N1	109.4 (4)
Cd1—O5—H5A	108 (5)	C9—C10—H10	125.3
Cd1—O5—H5B	110 (5)	N1—C10—H10	125.3
H5A—O5—H5B	109 (7)	N2—C9—C10	106.5 (4)
C11—N1—C10	104.7 (3)	N2—C9—H9	126.7
C11—N1—Cd1	125.3 (3)	C10—C9—H9	126.7
C10—N1—Cd1	129.8 (3)	C7—C6—C5	119.1 (3)
C11—N2—C9	106.7 (4)	C7—C6—C8	120.4 (3)
C11—N2—H2	129 (4)	C5—C6—C8	120.4 (3)
			
N1—Cd1—O1—C1	9.8 (3)	O2—C1—C2—C7	−156.1 (3)
O3^i^—Cd1—O1—C1	−133.6 (3)	O1—C1—C2—C7	24.1 (5)
O2^ii^—Cd1—O1—C1	138.2 (3)	O2—C1—C2—C3	24.1 (5)
O1^ii^—Cd1—O1—C1	92.8 (3)	O1—C1—C2—C3	−155.6 (4)
O4^i^—Cd1—O1—C1	−81.2 (3)	C7—C2—C3—C4	−2.3 (6)
N1—Cd1—O1—Cd1^ii^	−83.06 (11)	C1—C2—C3—C4	177.5 (4)
O3^i^—Cd1—O1—Cd1^ii^	133.56 (10)	C2—C3—C4—C5	2.6 (7)
O2^ii^—Cd1—O1—Cd1^ii^	45.34 (11)	C3—C4—C5—C6	−0.5 (7)
O1^ii^—Cd1—O1—Cd1^ii^	0.0	Cd1^i^—O3—C8—O4	4.2 (4)
O4^i^—Cd1—O1—Cd1^ii^	−174.03 (9)	Cd1^i^—O3—C8—C6	−176.6 (3)
O3^i^—Cd1—N1—C11	−24.3 (5)	C3—C2—C7—C6	−0.1 (5)
O2^ii^—Cd1—N1—C11	142.2 (3)	C1—C2—C7—C6	−179.9 (3)
O5—Cd1—N1—C11	59.3 (4)	C10—N1—C11—N2	−0.4 (5)
O1—Cd1—N1—C11	−111.6 (4)	Cd1—N1—C11—N2	175.6 (3)
O1^ii^—Cd1—N1—C11	172.5 (4)	C9—N2—C11—N1	1.0 (6)
O4^i^—Cd1—N1—C11	−18.7 (4)	C11—N1—C10—C9	−0.4 (5)
O3^i^—Cd1—N1—C10	150.6 (3)	Cd1—N1—C10—C9	−176.1 (3)
O2^ii^—Cd1—N1—C10	−42.9 (4)	C11—N2—C9—C10	−1.2 (5)
O5—Cd1—N1—C10	−125.8 (4)	N1—C10—C9—N2	0.9 (6)
O1—Cd1—N1—C10	63.3 (4)	C2—C7—C6—C5	2.1 (5)
O1^ii^—Cd1—N1—C10	−12.6 (4)	C2—C7—C6—C8	−178.0 (3)
O4^i^—Cd1—N1—C10	156.2 (4)	C4—C5—C6—C7	−1.8 (6)
Cd1^ii^—O2—C1—O1	0.1 (4)	C4—C5—C6—C8	178.3 (4)
Cd1^ii^—O2—C1—C2	−179.6 (3)	O4—C8—C6—C7	9.7 (5)
Cd1—O1—C1—O2	−103.8 (4)	O3—C8—C6—C7	−169.4 (3)
Cd1^ii^—O1—C1—O2	−0.1 (3)	O4—C8—C6—C5	−170.4 (4)
Cd1—O1—C1—C2	76.0 (4)	O3—C8—C6—C5	10.4 (5)
Cd1^ii^—O1—C1—C2	179.6 (3)		

Symmetry codes: (i) −*x*, −*y*+1, −*z*+1; (ii) −*x*, −*y*+1, −*z*+2.

### Hydrogen-bond geometry (Å, °)

**Table:**

*D*—H···*A*	*D*—H	H···*A*	*D*···*A*	*D*—H···*A*
O5—H5A···O2^iii^	0.84 (8)	2.07 (8)	2.809 (4)	146 (7)
O5—H5B···O3^iv^	0.87 (8)	1.96 (8)	2.786 (4)	158 (7)
N2—H2···O4^v^	0.85 (7)	2.00 (7)	2.854 (5)	175 (6)

Symmetry codes: (iii) *x*, *y*−1, *z*; (iv) *x*, *y*−1, *z*+1; (v) *x*+1, *y*, *z*.

## References

[bb1] Ma, S., Sun, D., Simmons, J. M., Collier, C. D., Yuan, D. & Zhou, H. C. (2008). *J. Am. Chem. Soc.* **130**, 1012-1016. 10.1021/ja0771639 18163628

[bb2] Sheldrick, G. M. (1996). *SADABS* University of Göttingen, Germany.

[bb3] Sheldrick, G. M. (2008). *Acta Cryst.* A**64**, 112–122. 10.1107/S0108767307043930 18156677

[bb4] Siemens (1996). *SMART* and *SAINT* Simemens Analytical X-ray Instruments Inc., Madison, Wisconsin, USA.

[bb5] Wang, J., Lin, Z., Ou, Y. C., Yang, N. L., Zhang, Y. H. & Tong, M. L. (2008). *Inorg. Chem.* **47**, 190–199. 10.1021/ic701721t 18069828

[bb6] Yaghi, O. M., Li, H., Davis, C., Richardson, D. & Groy, T. L. (1998). *Acc. Chem. Res.* **31**, 474–484.

